# Community-based respondent-driven sampling as a strategy for drug use surveillance in a large French urban area

**DOI:** 10.1186/s12954-023-00814-w

**Published:** 2023-06-29

**Authors:** Hélène Donnadieu, Catherine Quillet, Morgana D’Ottavi, Joëlle Castellani, Anne Debellefontaine, Sylvain Guichard, René Baglioni, Nicolas Langendorfer, Vincent Faucherre, Bertrand Hanslik, Edouard Tuaillon, Didier Laureillard, Nicolas Nagot

**Affiliations:** 1 Department of Addiction Medicine, Montpellier University Hospital, 80 Rue Augustin Fliche, 34090 Montpellier, France; 2 Pathogenesis and Control of Chronic and Emerging Infections, Etablissement Français du Sang, INSERM, University of Antilles-Guyane, University of Montpellier, 60 Rue de Navacelles, 34394 Montpellier, France; 3 AXESS, Harm Reduction Center, SOS Solidarités, 66 Avenue Charles Flahaut, 34090 Montpellier, France; 4 Association of Marginality and Drug Addiction (AMT), 10 Boulevard Victor Hugo, 34000 Montpellier, France; 5 Department of Infectious Diseases, Caremeau University Hospital, Place du Professeur Robert Debré, 30029 Nîmes, France

**Keywords:** Respondent-driven sampling survey, Drug use, Addiction care, Heroin, Methamphetamine, Access-to-care

## Abstract

**Background:**

Understanding drug use and behavior within the PWUD population is crucial to adapt harm reduction and prevention strategies, and provide improved addiction and medical treatment. However, in most countries such as France, the knowledge of drug use behaviors is likely biased as it originates from addiction centers which are attended by only an unknown proportion of PWUD. The objectives of this study were to describe drug use behavior in a population of active PWUD in the urban area of Montpellier, South of France.

**Methods:**

We implemented a community-based respondent-driven sampling survey (RDSS), a validated strategy to obtain a representative sample of a population, to recruit PWUD in the city. Adult individuals reporting frequent psychoactive drug use other than cannabis, with confirmation by urine test, were eligible. Beside HCV and HIV testing, trained peers interviewed participants on their drug consumption and behavior using standardized questionnaires. Fifteen seeds launched the RDSS.

**Results:**

During the 11 weeks of the RDSS, 554 actives PWUD were consecutively included. They were mostly men (78.8%), had a median age of 39 years, and only 25.6% had a stable living place. On average, participants consumed 4.7 (± 3.1) different drugs, and 42.6% smoked free-base cocaine. Unexpectedly, heroin and methamphetamine were consumed by 46.8% and 21.5% of participants, respectively. Among the 194 participants injecting drugs, 33% declared sharing their equipment.

**Conclusion:**

This RDSS highlighted a high consumption of heroin, crack and methamphetamine in this PWUD population. These unexpected results can be explained by low attendance to addiction centers, the source of drug use reports. Despite free care and risk reduction equipment in the city, sharing was very frequent among injectors, challenging the current program of harm reduction.

## Introduction

The best described patterns in drug use covering Europe are from the European Monitoring Center for Drugs and Drug Addiction (EMCDDA) [9]. In 2019, the EMCDDA reported an increase in the production and consumption of cannabis, cocaine and MDMA (3,4-methylenedioxy-*N*-methylamphetamine) in the region as well as an increase in poly-drug consumption. The indicators used in this report were mainly based on data collected from healthcare centers in several European countries.

As in most countries, the patterns of drug use in France have been collected either when people who use drugs (PWUD) seek care at an addiction care center, or through drug use complications reported by national hospitals (“addicto-vigilance”). Drug use practices and characteristics of these PWUD have been described in “*Coquelicot*”, an ANRS (French Agency for Research on AIDS and Viral Hepatitis) study from 2011 to 2013 which showed a high frequency of poly-consumption, a diversity of addictive practices, and cocaine as the substance the most consumed in France [23]. In addition, an increase of 52% in the use of crack cocaine between 2010 and 2017 has been reported [12].

Understanding drug use behavior within the PWUD population is thus crucial to adapt harm reduction and prevention strategies, offer better addiction care services and improve drug-related medical treatment [7]. Therefore, a large sample, representative of the marginalized population with frequent and poly-drug use appears necessary to document drug use behaviors and adapt harm-reduction programs. For this purpose, respondent-driven sampling survey (RDSS) has proven to be efficient [10]. As part of snowball sampling techniques, RDSS start with a non-random group of participants or “seeds” that are selected to represent the diversity of a target population. Each seed (and recruited participants) receives coupons which are then distributed to other PWUD in their network.

RDSS has successfully been used to recruit PWUD in many countries [1], mostly to provide estimates of the prevalence of HIV or hepatitis C (HCV) among people who inject drugs (PWID) [8, 14, 17] and collect additional data on drug use practices [3, 4, 15, 22, 24]. In Europe, there have been limited findings on the use of drugs and its consequences in the whole PWUD population. The objectives of this work were to describe drug use behavior in a population of active PWUD in the city of Montpellier (± 500,000 inhabitants), South of France, and understand the role of population-based data for the surveillance of drug use.

## Methods

We implemented a RDSS from September to November 2020*.* This study was approved by the French Ethical Committee South East V (#2018-A02667-48). Individual written informed consent was obtained from all participants prior to their participation in the study. Survey methods and results were reported according to the STROBE-RDS recommendations [25]. This study of drug use surveillance was integrated into a study aimed at facilitating access to anti-infective care for PWUD (ANRS-ICONE 95050).

### Study population

Active PWUD were eligible to participate in the study if they had a valid coupon, were aged 18 years or above, understood the research, were living in Montpellier Metropole (31 communes) and were not under guardianship. Active drug use was defined as self-report of psychoactive drug substance (PDS) consumption (with the exception of cannabis) for at least 10 times a month and at least once within the past three days (definition of regular use). Cannabis consumption alone was excluded in order to reach a rather poly-drug population with at-risk behaviors for transmission of infections. Thus, our study population had to resemble that usually attending in addiction care or harm-reduction centers. A drug urine test was then done to confirm the use of PDS.

### Study site and procedures

To build trust and earn confidence between the participants and the research team, most activities took place in a disused building, rehabilitated and equipped for the purpose of the study.

Peers (former or current PWUD) were hired and trained on all study procedures. Their role was to welcome participants, provide information on the study and carry out a face-to-face interview using a standardized questionnaire. They asked participants about their drug use and their access to care in the year preceding the study. The question of passage at least once in an addiction care or harm-reduction center was posed. In addition, they provided support and information on HIV, HBV, HCV and harm reduction related to drug use behavior.

We launched our RDSS via fifteen seeds who had each a network of at least five PWUD. These seeds were selected by local NGOs with social or prevention outreach activities targeting PWUD in Montpellier.

As per RDS principles, seeds are eligible PWUD who present diverse characteristics in terms of gender, age or district of residence. They must have a good network of PWUD to increase the likelihood of recruitment from this seed. Each seed received three coupons to distribute in their network.

When a participant showed a valid coupon at the research site, the inclusion criteria were checked, informed consent was obtained and a urine test was done using DOA-10 test cup (MB Biomedicals, Eschwege, Germany). Anthropometric measurements were collected to prevent duplicate participation. Peers carried out a face-to-face questionnaire including questions on socio-demographic characteristics, drug consumption, addiction behaviors and alcohol consumption [using the alcohol use disorder identification test consumption (AUDIT-C)] [21]. Once the questionnaire was completed, PWUD participated in harm-reduction sessions led by peers, and syringes and other sterile materials were made available for later drug consumption.

Rapid tests for HIV serology (INSTI VIH 1/2^®^, Nephrotek, Boulogne-Billancourt, France), hepatitis B virus (HBV) surface antigen (TOYO VHB^®^, Nephrotek, Boulogne-Billancourt, France) and HCV serology (TOYO VHC^®^, Nephrotek, Boulogne-Billancourt, France) were done by two trained nurses. For participants with a positive HCV rapid test, an Xpert HCV RNA^®^ and eventually a Fibroscan^®^ tests were immediately performed on-site, and if eligible, anti-viral treatment was prescribed by a physician. Peers provided support through all the process, including treatment.

As for the seeds, each participant received three coupons to be distributed in their network, a financial compensation of 50 Euro for their participation and an additional 20 Euro per returned coupon.

### Sample size calculation

In the absence of any RDS survey implemented in France before this study, we could not precisely estimate the number of participants to recruit. However, we aimed at recruiting at least 400 PWUD.

### Data analysis and statistical methods

Participant characteristics (e.g., gender, nationality, and sources of revenue), drug use (self-declaration of consumption over the past month, and detection in urine), the administration route (e.g., sniffed and injected) and injection materials and practices for PWID were described using counts and percentages for categorical variables, and means with their standard deviation or medians with the interquartile range (IQR) for continuous variables, depending on their distribution. For Nationality, only the five most represented countries are described. For the AUDIT-C test, a score, from 0 to 12 was calculated. Alcohol was considered at risk of alcohol misuse when the score was ≥ 3 for women and ≥ 4 for men, and at risk of alcohol dependence when the score was ≥ 10 [21].

RDSS diagnostics were checked including homophily, and the number of waves before reaching equilibrium for key variables.

All statistical analyses were performed using Stata v16.1 (Stata Corp LP, College Station, USA). The RDS Coupon Manager tool (V3.1) was used to handle coupons and the RDS Analysis Tool (RDSAT V7.1) to estimate weights and verify diagnostic measures.

## Results

**Fig. 1:**
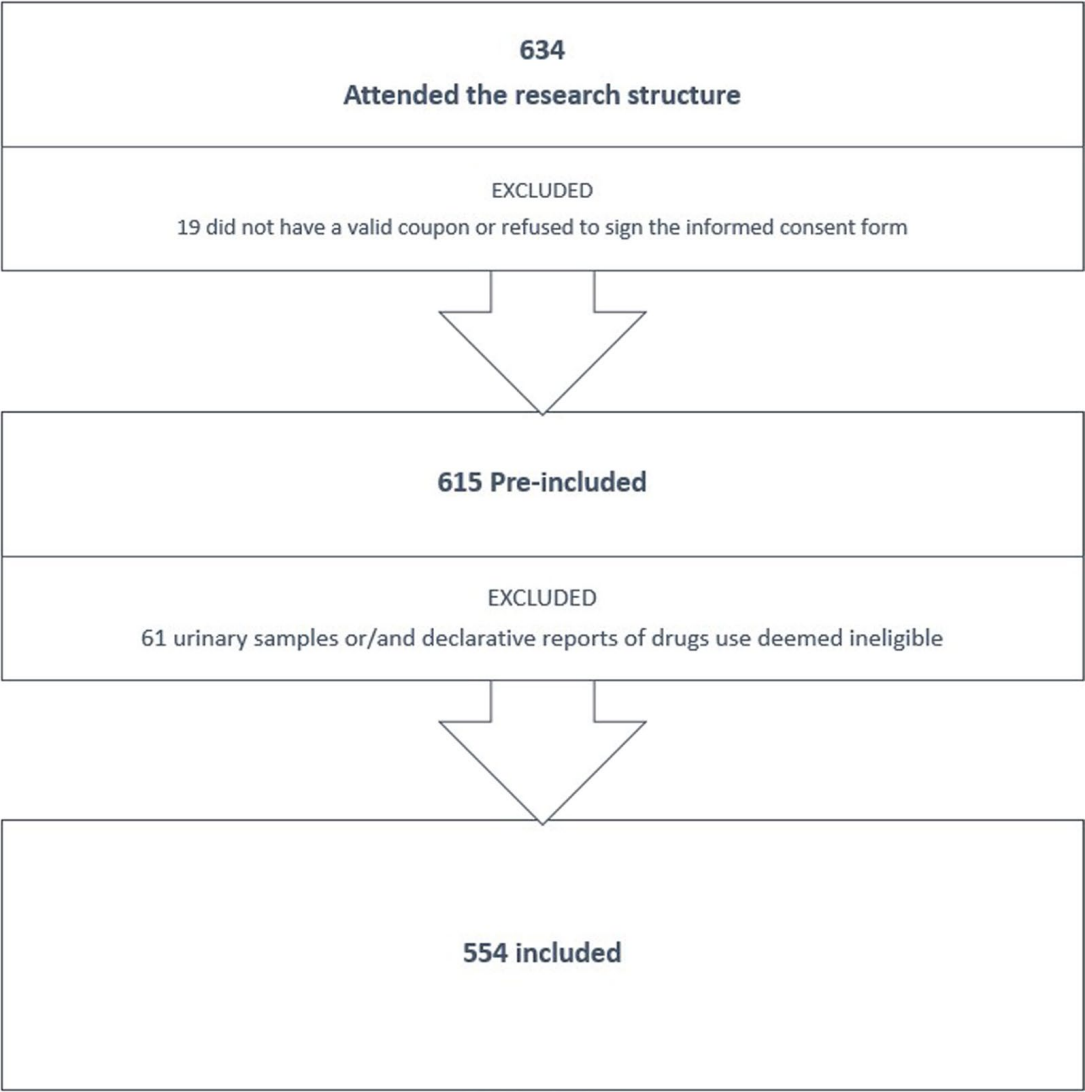
Inclusion of the participants in the study

Within the 11 weeks of recruitment, 634 candidates came to the research site (Fig. 1). Nineteen were excluded because they did not have a valid coupon or refused to sign the informed consent form, and 61 did not meet the eligibility criteria of drug consumption. In total, 554 active PWUD were included in the study.

### Characteristics of the participants

Participants were mostly men (78.8%) with a median age of 39 years [IQR: 33 to 46] (Table 1). Overall, 76.0% were French but 29 other nationalities were represented in the sample (Table 1). Among participants, 48.9% were living alone and 74.4% had no stable living place. Only 12.8% had a professional activity as their main source of income, 26.2% have ever been hospitalized in a psychiatric ward, of which 87.6% in France. Lifetime incarceration was reported by 52.2% of the participants, including 81.7% in France. Rapid diagnostic tests revealed that 3.1% of participants were HIV positive, 1.4% had detectable AgHBs, 32.6% had a positive HCV serological test and 8.8% of all enrolled participants had a detectable RNA HCV.

**Table 1:** Characteristics of PWUD enrolled in the RDSS

Category	Sub-category	*N* (%)
Gender	Men	437 (78.8)
	Women	115 (20.8)
	Other	2 (0.4)
Age, median [IQR]		39 [33 to 46] min 18, max 65
Nationality	French	421 (76.0)
	Georgian	24 (4.3)
	Czech	19 (3.4)
	Moroccan	17 (3.1)
	Algerian	13 (2.3)
Living situation	Isolated	271 (48.9)
	Cohabiting	83 (15.0)
	With family	61 (11.0)
	With friends	139 (25.1)
Dwelling	Stable	142 (25.6)
	Temporary	185 (33.4)
	Squat	125 (22.6)
	Homeless	102 (18.4)
Source of income*	Professional activities	71 (12.8)
	Benefits/social minimums	348 (62.8)
	Begging	130 (23.5)
	Family	36 (6.5)
	Illegal activities	81 (14.6)
Health insurance	Regular scheme	188 (33.9)
	PUMA or AME	286 (51.6)
	None	80 (14.4)
Enrolled in an addiction care center	Yes	148 (26.7)

*AME* Aide médicale de l’Etat (Medical financial assistance from the government), *IQR* Interquartile range, *PUMA* Protection Universelle Maladie (universal health insurance), *PWUD* people who use drugs, *RDSS* Respondent-driven sampling survey

*Participants may be in more than one category

The participants’ engagement in care and care-seeking behaviors were generally low with 59.0% having seen a general practitioner, 32.9% had been to a low-threshold harm-reduction center (CAARUD), and 26.7% had been to an addiction care center (CSAPA). At least once in the year preceding the study.

### RDS diagnostics

Overall, the RDS diagnostics were satisfactory. The number of waves to reach equilibrium for gender, HCV serology and the type of PWUD (injecting drugs or not) was two, three and three respectively. Using a predefined threshold of 0.3, homophily was reached for gender, HCV serology and was borderline for the type of PWUD.

411 PWUD (74.2%) reported consuming alcohol during the last year. Among all participants, 73/115 women (63.5%) and 304/437 men (69.6%) were at risk of alcohol misuse. Among those who were consuming alcohol, 213 participants (51.8%), irrespective of gender, were at risk of alcohol dependence. Almost all PWUD (97.3%) were active smokers of tobacco, and roughly three-fourths (74.7%) had smoked cannabis over the past month at the time of the questionnaire.

Study participants had, on average, consumed 4.7 (± 3.1) different PDS during the past month. Opioids (78.5%) were the PDS the most consumed. A large proportion (46.8%) of participants declared heroin consumption in the past month (Table 2). Cocaine (73.1%) and free-base cocaine (42.6%) were also commonly taken. Among the psychostimulants widely consumed, methamphetamine was mentioned by 21.5%. Among all participants, 12.8% (71/554) consumed all following three substances: opioids, cocaine and methamphetamine. Drugs diverted from their intended medical use (i.e., ORT, Ketamine, GBL/GHB, sleeping pills and methylphenidate) were largely inappropriately taken (Table 2).

**Table 2:** Psychoactive drug substances consumed in the past month and administration route declared by PWUD

Category	Sub-category	At least once in the last month*	Main administration route*	
		*N* (%)		*N* (%)
Opioids	Heroin	259 (46.8)	Sniffed	176 (68.0)
			Smoked	68 (26.3)
			Injected	53 (20.5)
	Opium	48 (8.7)	Smoked	25 (52.1)
			Sniffed	14 (29.2)
	Rachacha**	20 (3.6)	Ingested	14 (70.0)
	Synthetic opioids***	24 (4.3)	Sniffed	9 (37.5)
Opioids diverted from their medical use	Methadone	194 (35.0)	Ingested	185 (95.4)
	Buprenorphine	176 (31.8)	Ingested	124 (70.5)
			Sniffed	49 (27.8)
			Injected	46 (26.1)
	Analgesic opioids	176 (31.8)	Ingested	94 (53.4)
			Injected	80 (45.5)
Cocaine		405 (73.1)	Sniffed	234 (57.8)
			Injected	142 (35.1)
Free-base cocaine		236 (42.6)	Smoked	230 (97.5)
Stimulants	Amphetamines, MDMA	214 (38.6)	Sniffed	103 (48.1)
			Ingested	145 (67.8)
	Methamphetamines	119 (21.5)	Sniffed	57 (48.0)
			Injected	38 (31.9)
			Smoked	25 (21.0)
Misused prescription drugs	Ketamine	93 (16.8)	Sniffed	74 (79.6)
	GBL/GHB	19 (3.4)	Ingested	15 (78.9)
	Sleeping pills	127 (22.9)	Ingested	118 (92.9)
	Methylphenidate	81 (14.6)	Injected	65 (80.2)
Hallucinogenic		94 (17.0)	Ingested	89 (94.7)
Solvents		52 (9.4)	Inhaled	35 (67.3)
			Sniffed	17 (32.7)
Cathinone		19 (3.4)	Sniffed	9 (47.4)
			Injected	5 (26.3)

*GBL* y-Butyrolactone, *GHB* Gamma-Hydroxybutyric acid, *MDMA* 3,4-Methylenedioxymethamphetamine, *PWUD* People who use drugs

*Participants may be in more than one category

**Homemade paste obtained by decoction of poppy heads

***Apache, arrache, China white, China girl, Dance fever, Drop Dead, Flatline, Goodfella, Great Bear, héroïne de synthèse, Jackpot, Lollipops, Murder 8, rach, Perc-o-Pops, Poison, TNT…

The results of the urine sample analysis are detailed in Table 3. Substance detection in the urine sample confirmed a primary consumption of opioids, cocaine and misused medications.

**Table 3:** Psychoactive drug substances found in the urine sample among PWUD

Category	All* (*N *= 554)	PWID* (*N *= 194)
	*N* (%)	*N* (%)
COC (cocaine)	346 (62.5)	110 (56.7)
MOR (heroin, morphine)	251 (45.3)	103 (53.1)
MDMA (Ecstasy)	30 (5.4)	8 (4.1)
MET (methamphetamine)	46 (8.3)	25 (12.9)
AMP (amphetamines)	74 (13.4)	21 (10.8)
MTD (methadone)	192 (34.7)	87 (44.8)
BUP (buprenorphine)	165 (29.8)	86 (44.3)
KET (ketamine)	25 (4.5)	7 (3.6)
MDP (methylphenidate)	79 (14.3)	48 (24.7)
MCAT (cathinone)**	20 (3.6)	10 (5.2)

*PWID* People who inject drugs, *PWUD* People who use drugs

*Participants may be in more than one category

**MCAT was only tested in men who report taking it in a context of chemsex (*N *= 148 among whom 50 were injectors)

### (Mis)use of opioid replacement therapy (ORT)

Three hundred eighty-two participants (69.0%) either declared taking methadone (MTD) and/or buprenorphine (BUP) or had them in their urine sample. Most mentioned consuming them both as ORT and/or diverted from their medical use (*N *= 319; 83.5%). MTD/BUP use following medical prescription was mentioned by 3.9% (*N *= 15). Among those who consumed MTD/BUP within the past three days (i.e., positive urine sample), MTD/BUP had been obtained by prescription for 198 (59.3%) participants. The main places, mentioned by MTD/BUP consumers (following medical prescription or recreationally), to receive a prescription was an addiction care center (CSAPA) (27.5%), a general practitioner (26.6%) and a specialty harm-reduction care center (known as an *acceuil bas seuil* in French) (6.6%). Other PWUD bought this treatment on the street (“*black market*”).

### Drugs practices and equipment sharing

Overall, 194 participants (35%) reported injecting drugs at least once in the previous month. Among them, 142 (73.2%) consumed by injection cocaine then analgesic opioids (90, 46.4%) and Methylphenidate (65, 33.5%). Heroin by injection concerned 53 (27.3%) of participants who injected in the last month then Buprenorphine (46, 23.7%) and Methamphetamines (38, 14.4%).

Most PWID obtained their injection equipment from harm-reduction centers (66.5%) or from pharmacies (62.9%), and 33% said they shared their injection equipment with other drug users.

Among PWID, they shared their needles (18.0%), syringes (21.6%), filter or cotton (22.7%) or their cup (21.6%). Only 30.9% systematically washed their hands before drug injection and 49.5% stored their syringes or needles in purpose-specific containers. Among people who use other modes of consumption than injecting, 203 (45.9%) mentioned having shared a straw and 204 (46.2%) a pipe for free-base crack/cocaine consumption in the past six months. Having attended a harm-reduction center during the previous year did not improve these injection practices.

## Discussion

The use of the RDSS approach together with peers trained for addiction interviews and urine test made it possible to accurately understand drug use and behavior of the mainly marginalized PWUD population with frequent and poly-drug use.

Our findings contrast with our knowledge before the study. Within 11 weeks, 554 PWUD participated in this study, which confirms that RDSS are highly efficient to recruit participants from this largely marginalized population.

The socio-demographic characteristics of the RDSS participants confirmed a high level of social marginalization and vulnerability of PWUD [11, 19].

Three keys’ findings emerged from this innovative population-based study that were unknown before its implementation. First, heroin/opioid consumption (both self-declared and/or detected in urine) was much higher than previously estimated in studies conducted at addiction care facilities [23]. Our study showed that almost half of the participants reported using heroin at least once in the last month (mainly via sniffing), confirmed by a urine test. Second, only a quarter of our PWUD were enrolled in an addiction care center, and a third of PWID shared their injection equipment with other drug users. Finally, our study confirms the high rate of poly-consumption by drug users and the spread of cocaine and free-base cocaine consumption in France [16].

In 2019, the EMCDDA showed an increase in the use of synthetic opioid and heroin [9]. Both drugs were remaining the most common opioid/opiate on the European drug market although the number of heroin addicts admitted for treatment have declined in most countries in Europe [9]. Brunt et al. [6] collected syringes from automatic injection kit dispensers on streets or at harm-reduction services in seven European cities in 2017 and 2018. The most commonly detected substances were cocaine (31%), heroin (24%), synthetic cathinones (17%), buprenorphine (17%) and amphetamines (17%). In Paris, they found that heroin was detected only in 17% (2017) and 20% (2018) of the analysis of syringes while synthetic cathinones (51% in 2018) and cocaine (49% in 2018) were the most commonly found substances. Using the data collected from addiction care centers in France, the *Observatoire Français des Drogues et des Toxicomanies* (OFDT) known as the French Monitoring Center for Drugs and Drug Addiction, described a stability in the use of heroin among those aged 18 to 64 years old (1.3%) and a decrease in heroin initiation among those aged 17 years and above from 2014 to 2017 (0.7%), while heroin-related seizures by the police increased [16].

Drug use patterns in France were studied by the ANRS (*Coquelicot* survey—2011–2013) [23]. This very large survey, involving several French cities, enrolled PWUD from addiction care centers and harm-reduction facilities and used face-to-face interviews conducted by staff specialized in drug use behavior. Our population-based estimates differ from those originating from these addiction/harm-reduction centers in several ways. We noted differences that are firstly explained by the use of peers for interviews, which allowed us to reduce desirability bias and underreporting drug use. Second, the ANRS-*Coquelicot* study only enrolled PWUD from addiction care centers which may not be representative of the entire PWUD population as those PWUD have probably reduced their drug consumption to improve their health. Finally, the Coquelicot study was not carried out in Montpellier, and we know that wide disparities can exist between cities.

Despite free care for all PWUD at addiction care centers in France, only a quarter of our participants reported having been to such centers over the previous 12 months, and only a third reported having attended a harm-reduction structure. As mentioned before, the strength of RDSS and peer involvement made it possible to reach PWUD who do not typically attend addiction care centers.

The month prior the study, 194 participants reported at least one injection for which injection materials were obtained mainly in harm-reduction centers and/or in pharmacies. However, although injection materials can be obtained free of charge, not all drug users accessed such centers. This is mainly due to barriers such as stigma, misinformation and the absence of motivation. Although NSP and injection assistance have shown to be effective in the reduction of infectious diseases, crime, overdose, and mortality among PWUD [26], our results suggest that the French harm-reduction program has a too limited coverage. This situation may challenge the achievement of HCV elimination in this population. Raising awareness of this situation and developing innovative outreach strategies are urgently needed. For example, educational programs on safer injection practices can reduce syringe sharing and the appearance of cutaneous abscesses [20]. PWID enrolled in these harm-reduction programs receive advice on clean injection techniques and in a few cases, when drug consumption rooms are available, they can inject on-site with sterile materials. However, as for NSP, these educational programs are not sufficiently widespread in France, and in fact, only two drug consumption rooms are available in the whole country. The relevance of such programs in France is currently being studied [2]. Historically, addiction care (CSAPA) and harm-reduction programs (CAARUD) were carried out in different places. We could imagine increasing training and budgets for harm reduction in CSAPAs, in order to systematically offer this notion in the care of PWUD. Finally, the distribution of "Stop addiction" (“*Haltes addiction*”) structures on the French territory could minimize at-risk behaviors when using drugs.

Finally, our study confirms the effectiveness and efficiency of peers for activities among marginalized populations. Peers involvement has been reported as the most successful method to reach PWUD, screen them for HIV and HCV, engage them in care and provide them with prevention tools [5, 18]. It also plays a key role in harm reduction, positive health-related behavior and proper treatment adherence and compliance [13].

Our study had several strengths. First, a urine test was done to confirm the use of drugs which is not often done in studies in this field. Declarative addictological data are often criticized, but here we see that users declared consumptions that are consistent with what was found in their urine samples. These results are important and will make it possible in the future to interpret with less difficulty the drugs declared by users.

Second, our findings, which include PWUD not enrolled in addiction care or harm-reduction centers, and the use of RDSS have major public health implications as they challenge the current methods of measurement and surveillance of drug use in France and Europe. Furthermore, contrary to popular belief, even if care is free of charge, coverage for medication-assisted treatment is not large. Finally, they also highlight the need to improve current addiction prevention and care programs in France.

In terms of limitations, our study was first a single-site study which represented drug use at a given time and only in a specific city. Despite the interviews being carried out by peers, desirability bias and underestimation of alcohol and PDS use based on self-declaration consumption could not be completely ruled out since financial compensation was an important incentive for participation in the study.

In conclusion, our study highlighted a much higher than expected use of heroin and methamphetamine among PWUD in a large urban area of France. It also raised concern on the poor population coverage of addiction and harm-reduction centers which prompt the need for innovative strategies. As a consequence, equipment sharing stands high among PWUD, questioning the future achievement of HCV elimination in this population. However, further research in other large French cities may be needed to confirm our findings.

## Data Availability

The data that support the findings of this study are available from the corresponding author, HDR, upon reasonable request.
